# Comparative Analysis of Olfactory Receptor Repertoires Sheds Light on the Diet Adaptation of the Bamboo-Eating Giant Panda Based on the Chromosome-Level Genome

**DOI:** 10.3390/ani13060979

**Published:** 2023-03-08

**Authors:** Chuang Zhou, Yi Liu, Guangqing Zhao, Zhengwei Liu, Qian Chen, Bisong Yue, Chao Du, Xiuyue Zhang

**Affiliations:** 1 Key Laboratory of Bioresources and Ecoenvironment (Ministry of Education), College of Life Sciences, Sichuan University, Chengdu 610064, China; 2 Key Laboratory of Sichuan Province for Fishes Conservation and Utilization in the Upper Reaches of the Yangtze River, Neijiang Normal University, Neijiang 641000, China; 3 Baotou Teachers College, Baotou 014060, China

**Keywords:** giant panda, diet adaptation, genome-wide analysis, chromosome-level, olfactory receptor, odorant recognition

## Abstract

**Simple Summary:**

Olfaction in animals plays important roles in many aspects, such as food recognition, mate detection and risk avoidance and social communication. Compared to other Ursidae species, the obligate bamboo feeder, giant panda, shows special diet, and how the diet transformation affects the olfactory system remains little known. In this study, we identified the olfactory receptor (OR) genes of the giant panda based on the chromosome-level genome and conducted comparative analysis of OR genes among Ursidae species. The giant panda had 639 OR genes, and chromosome 8 had the most OR genes. The giant panda had 31 unique OR gene subfamilies (containing 35 OR genes), of which 10 genes were clustered into 8 subfamilies with 10 known human OR genes (OR8J3, OR51I1, OR10AC1, OR1S2, OR1S1, OR51S1, OR4M1, OR4M2, OR51T1 and OR5W2). Compared to other Ursidae species, the giant panda lacked OR genes similar to OR2B1, OR10G3, OR11H6 and OR11H7P, which may be related to the diet transformation from carnivore to herbivore. Hence, these results may shed light on the olfactory function and variation of the giant panda.

**Abstract:**

The giant panda (*Ailuropoda melanoleuca*) is the epitome of a flagship species for wildlife conservation and also an ideal model of adaptive evolution. As an obligate bamboo feeder, the giant panda relies on the olfaction for food recognition. The number of olfactory receptor (OR) genes and the rate of pseudogenes are the main factors affecting the olfactory ability of animals. In this study, we used the chromosome-level genome of the giant panda to identify OR genes and compared the genome sequences of OR genes with five other Ursidae species (spectacled bear (*Tremarctos ornatus*), American black bear (*Ursus americanus*), brown bear (*Ursus arctos*), polar bear (*Ursus maritimus*) and Asian black bear (*Ursus thibetanus*)). The giant panda had 639 OR genes, including 408 functional genes, 94 partial OR genes and 137 pseudogenes. Among them, 222 OR genes were detected and distributed on 18 chromosomes, and chromosome 8 had the most OR genes. A total of 448, 617, 582, 521 and 792 OR genes were identified in the spectacled bear, American black bear, brown bear, polar bear and Asian black bear, respectively. Clustering analysis based on the OR protein sequences of the six species showed that the OR genes distributed in 69 families and 438 subfamilies based on sequence similarity, and the six mammals shared 72 OR gene subfamilies, while the giant panda had 31 unique OR gene subfamilies (containing 35 genes). Among the 35 genes, there are 10 genes clustered into 8 clusters with 10 known human OR genes (OR8J3, OR51I1, OR10AC1, OR1S2, OR1S1, OR51S1, OR4M1, OR4M2, OR51T1 and OR5W2). However, the kind of odor molecules can be recognized by the 10 known human OR genes separately, which needs further research. The phylogenetic tree showed that 345 (about 84.56%) functional OR genes were clustered as Class-II, while only 63 (about 15.44%) functional OR genes were clustered as Class-I, which required further and more in-depth research. The potential odor specificity of some giant panda OR genes was identified through the similarity to human protein sequences. Sequences similar to OR2B1, OR10G3, OR11H6 and OR11H7P were giant panda-specific lacking, which may be related to the transformation and specialization from carnivore to herbivore of the giant panda. Since our reference to flavoring agents comes from human research, the possible flavoring agents from giant panda-specific OR genes need further investigation. Moreover, the conserved motifs of OR genes were highly conserved in Ursidae species. This systematic study of OR genes in the giant panda will provide a solid foundation for further research on the olfactory function and variation of the giant panda.

## 1. Introduction

Olfactory receptors are encoded by the OR genes and synthesized by olfactory neuronal cells. They have 7 transmembrane structures of G-protein coupled receptors with an average length of about 310 amino acids. They are widely found in vertebrates and first discovered in rodents in 1991 [1]. In vertebrates, OR genes can be divided into 10 categories (α, β, γ, δ, ε, ζ, η, θ, κ, λ) according to phylogenetic relationships. Six categories (α, β, γ, δ, ε, ζ) belong to Class-I, and four categories (η, θ, κ, λ) belong to Class-II [2]. Class-I genes were first found in fish and can recognize odor molecules that were soluble in water. Class-II genes were mainly found in mammals and can identify odor molecules dissolved in the air [3]. The OR gene family is composed of many different subfamilies which serve more complex functions. Compared with other genes, OR genes have extremely remarkable features. Its coding region sequence length is about 1 kb, which has no introns. Introns usually appear in the 5′ untranslated region of the gene, and the non-coding exons in 5′ can lead to alternative splicing and regulate the expression of OR genes to produce different olfactory receptor mRNA subtypes [4]. Each OR gene expresses an OR receptor, and the diversity of ORs is determined by the diversity of OR genes. There is no one-to-one relationship between OR and odor molecules; on the contrary, one odor molecule can be recognized by multiple ORs, and one OR can recognize multiple odor molecules [5].

In the mammalian genome, OR genes account for the largest proportion, accounting for about 3–5% of the total genome, so it is also called the olfactory subgenome [6]. Although mammals have the largest number of OR genes, they retain the smallest number of gene families. Bioinformatics analysis of different mammalian genome sequences shows that the number of OR genes varies greatly among different species. Obviously, the number of OR genes is affected by the environment of each species. For example, in the process of cetaceans from terrestrial to aquatic, there was a decrease in the number of Class-I genes first, then a decrease in the number of Class-II genes, and an echolocation system adapted to the water environment evolved [7]. In addition, the echolocation system also exists in bats, but scholars have found that the OR genes of bats and their echolocation systems do not have a sensory compensation mechanism, and their olfaction is closely related to their special lifestyle [8]. A mouse has about 1200 functional OR genes in the genome [9]. However, primates usually have a smaller number of OR genes; there are about 400 functional OR genes in the human genome [10]. Chimpanzees have almost the same number of functional OR genes as humans, although macaques have considerably fewer OR genes [11]. These findings all reflect that primates will rely more on sight compared to the sense of olfaction. However, the reason and the timing for the “loss” of OR genes are still unclear in the evolution of animals.

**Figure 1:**
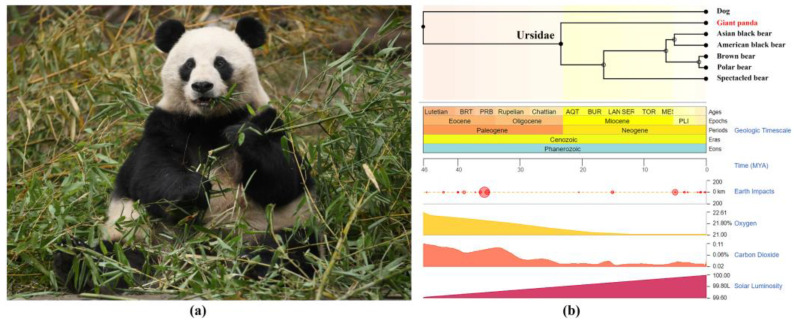
The bamboo-eating giant panda within the Ursidae. (**a**) A photo of the giant panda was taken by Bo Luo. (**b**) The phylogenetic position of the giant panda from TimeTree (http://timetree.org/, accessed on 1 January 2022).

The giant panda (Figure 1), one of the most endangered mammals in the world, is still under threat due to strong pressure from the environment and humans. In 2012, the Fourth National Survey of Giant Pandas showed that the population of giant pandas was estimated to be 1864 across 25,349 km^2^ of habitat [12]. The giant panda’s conversion to an herbivorous diet has produced unique adaptations in many aspects, for instance, pseudothumbs and a low energy metabolism rate, to accommodate to low-nutrient and low-energy consumption food [13]. The comparative genomic analysis revealed that the giant panda has similar genetic characteristics to carnivores in terms of olfaction [14]. The giant panda can use smell cues in urine and body odor to distinguish relatives from non-relatives. Compared with the mother’s smell, the daughters prioritized the research on the smell of unrelated adult pandas, while the mother spent more time studying the smell of the female pandas matched with her age, rather than the smell of her daughters [15]. Giant pandas smear perianal gland secretions or urine on trees and rocks to form smell marks, and the place where smell marks are concentrated is usually their estrus field. A total of 951 chemical components were identified from the giant panda’s odor glands, urine, vaginal secretions and odor markers using mass spectrometry. The odor markers of the two sexes contain similar chemicals, but at different concentrations. Specifically, males had a lot of short-chain fatty acids [16]. Some genes located in the cat, giant panda and dog evolutionary breakpoint regions (EBRs) were enriched in olfaction, indicating that the evolution of olfactory system in the giant panda may be related to chromosome rearrangement events [17]. Some olfactory proteins interact with conjectural pheromones, and some chemical components may be useful for successful reproduction of giant pandas through typically complex chemical communication systems [18]. The giant panda can be considered as an attractive animal olfaction model to perform a study, due to its transformation and specialization from carnivore to herbivore. With the development of third-generation sequencing technology, the latest version of the giant panda’s genome has reached the chromosome level [17]. In this study, we analyzed the composition, specificity, phylogeny and conserved motifs of giant panda OR at the chromosome level of the genome. This research can provide basic information for further research on the olfactory system of the giant panda.

## 2. Materials and Methods

### 2.1. Genome Data Collection

The complete genomes of the giant panda, spectacled bear, American black bear, brown bear, polar bear and Asian black bear were downloaded from NCBI (https://www.ncbi.nlm.nih.gov/, accessed on 1 January 2022) (giant panda: GCF_002007445.1, spectacled bear: GCA_018398825.1, American black bear: GCF_020975775.1, brown bear: GCF_003584765.2, polar bear: GCF_000687225.1, and Asian black bear: GCA_009660055.1).

### 2.2. OR Gene Identification

As in previous studies, an homologous search method was used to detect OR genes in the giant panda genome [19]. Full-length sequences of mammalian OR genes were collected manually with the keyword “olfactory receptor” at NCBI. Using the obtained OR genes as a query, a TBLASTN search [20] was performed on these six whole genomes, and the E value was 1e-10. The best matches with the lowest E value and the longest alignment were retained. Using Solar [21] combined fragment sequence and GeneWise [22] to predict gene structure. The hmmscan program of the HMMER3 software package (http://hmmer.janelia.org, accessed on 1 January 2022) was used to search for OR genes in the set of translated predicted OR proteins. Finally, the identified OR genes were classified into three categories: functional genes, partial genes and pseudogenes. Pseudogenes were the OR genes with stop codons and/or frameshifts in the ORF. Of the remaining identified OR genes, functional genes were at least 250 amino acids in size, and the rest were considered to be partial genes.

### 2.3. Phylogenetic Analysis and Classification

The amino acid sequences of functional OR genes in the above six animals were aligned by using MAFFT 7 (https://mafft.cbrc.jp/alignment/software/, accessed on 1 January 2022) with default parameters. The best-fit model (JTT + R10) of IQ-TREE v1.6.10 [23] was used to conduct 1000 bootstrap replications through the maximum likelihood (ML) method to construct an unrooted phylogenetic tree. Before phylogenetic analysis, we used the ML algorithm in MEGA7 to check the multiple alignments of functional OR gene amino acid sequences [24]. CD-HIT software v4.8.1 [25] was used to perform multi-species functional OR gene clustering analysis, which was based on the functional OR protein sequences of six species. OR genes can be further divided into families (homology > 40%) and subfamilies (homology > 60%) according to amino acid sequence homology [6]. According to the results of phylogenetic analysis and clustering analysis, the identified functional OR genes were finally divided into families and subfamilies.

### 2.4. Chromosomal Distribution and Motifs Analysis

TBtools was used to plot the gene location of functional OR genes on chromosomes, which visualized the OR gene organizations better. The Multiple Expectation Maximization Motivation (MEME) program [26] was employed to generate sequence logos for subsequent identification of conserved motifs in the functional OR amino acid sequences. We identified the top five conserved motifs, with a motif length ranging from 5 to 50. Potential N-glycosylation sites were predicted using NetNGlycserver [27].

## 3. Results

### 3.1. Composition of OR Gene Repertoires

**Figure 2:**
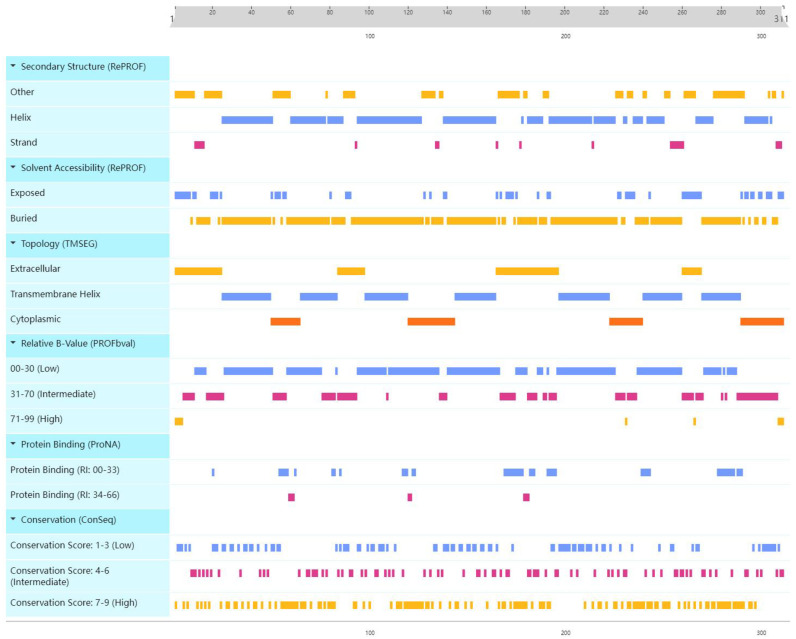
Structure information of ORs of the giant panda.

**Figure 3:**
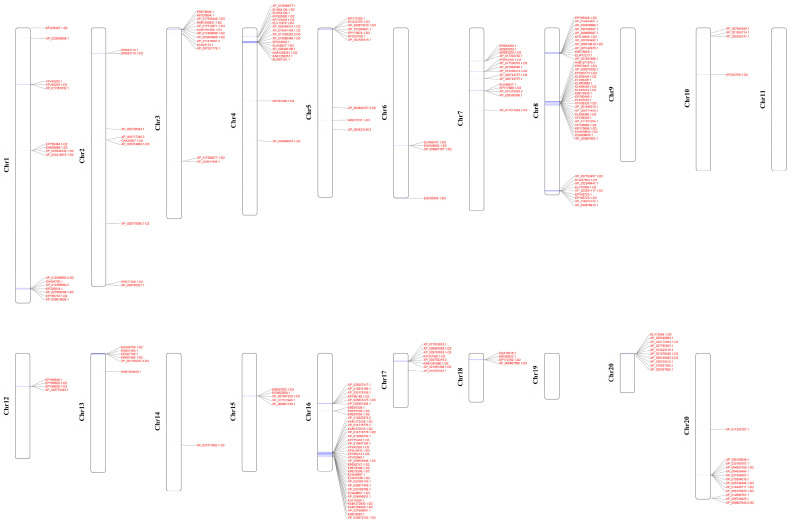
Chromosomal distribution of the functional OR genes of the giant panda.

The OR structure information of the giant panda was obtained by PredictProtein (https://predictprotein.org/, accessed on 1 January 2022) and revealed in Figure 2, containing solvent accessibility, secondary structure, transmembrane helices and conservation. The total number of OR genes in the giant panda was 639, including 408 functional genes, 94 partial genes and 137 pseudogenes. The percentage of functional genes in the American black bear was the highest among these six species (Table 1), while the percentage of functional genes in the giant panda was only 63.85%, just a little more than the lowest one: the spectacled bear (*n* = 60.04%). It is worth noting that the total number of OR genes and functional genes in the Asian black bear was apparently higher than other five species. In addition, the percentage of OR pseudogenes in the Asian black bear was the lowest. These data suggested that the smell sense of the Asian black bear possibly developed well. The number and percentage of OR pseudogenes in the giant panda were both the highest among these six species. In the other five species, the percentage of OR pseudogenes ranged from 11.99% to 18.04%. These genes have lost the ability to encode functional proteins during evolution, which is a relic of evolution. However, studies [28] have shown that some OR pseudogenes still have expression activity, and their products may participate in the regulation of functional OR gene expression. As for the partial genes, the proportion varied widely in each species. The spectacled bear was the highest (*n* = 23.67%) while the American black bear was the lowest (*n* = 2.76%). Among these 639 OR genes of the giant panda, 222 genes were distributed on 18 chromosomes (Figure 3). Remarkably, the genes were distributed in clusters on chromosomes. Generally, if the distance between two OR genes was less than 1 Mb, the genes could be clustered to one cluster. Furthermore, chromosome 8 (*n* = 44) and chromosome 16 (*n* = 38) had the most functional OR genes. Moreover, among the remaining chromosomes, there were no more than 20 functional OR genes on any chromosome (Table 2). However, no OR genes had been detected on chromosomes 9, 11 and 19. This may be because the fragments that many OR genes locate were not assembled to the chromosome level.

### 3.2. Classification of OR Gene Repertoires

**Figure 4:**
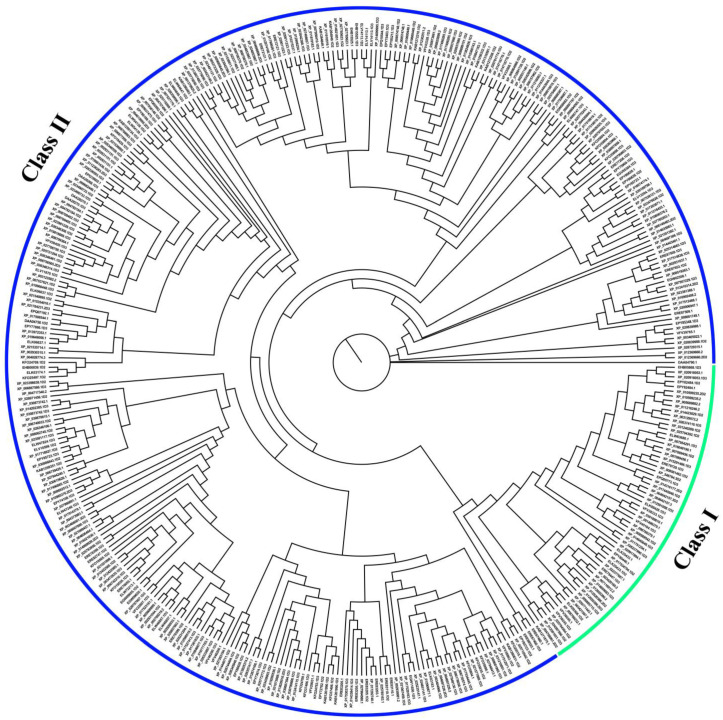
The phylogenetic tree of functional OR genes in the giant panda.

**Figure 5:**
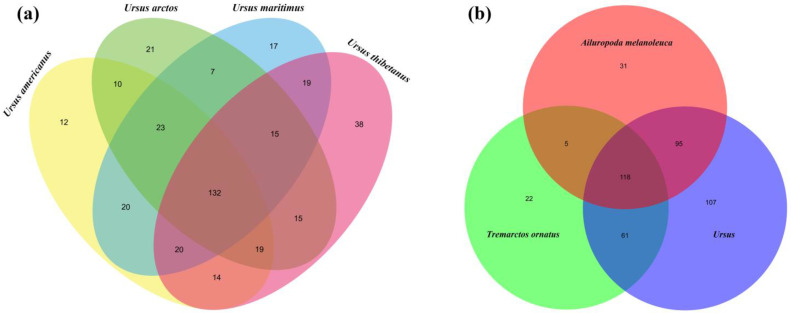
Comparison of OR gene clusters (**a**) between four species from genus *Ursus* (**b**) between three different genera from family Ursidae.

Mammals have two types of OR genes: Class Ⅰ and Class Ⅱ, which were usually clustered into two independent branches on the phylogenetic tree, and they may have different origins [29]. Generally, only Class Ⅰ genes exist in the genomes of aquatic animals, while there are two types of genes in the genomes of terrestrial animals, and most of the Class Ⅰ genes have specific functions. In our phylogenetic tree, the blue branch was Class II, and the green branch was Class I (Figure 4). In terms of quantity, 345 functional OR genes were clustered to Class Ⅱ, while only 63 functional OR genes were clustered to Class Ⅰ. Class Ⅱ genes were obviously more than Class Ⅰ genes, and this phenomenon exists in most mammals. Furthermore, according to the phylogenetic analysis of OR genes and OR protein sequence similarity, we divided functional OR genes into families and subfamilies. The Asian black bear had the largest amount of OR gene subfamilies; however, the spectacled bear had the least (Table 1). The giant panda has 639 OR genes, which can be divided into 39 families and 248 subfamilies. The average number of genes in each subfamily was less than 2 (1.64), indicating that the OR gene family of the giant panda had rich sequence diversity. In addition, our clustering analysis of OR gene subfamily showed that there were a total of 382 clusters in four *Ursus* species (American black bear, brown bear, polar bear and Asian black bear) and 439 clusters in the six species from three genera (*Ailuropoda melanoleuca, Tremarctos ornatus* and *Ursus*). They were relatively conservative in the evolutionary process and may be responsible for the identification of some common odors to maintain the basic olfactory ability of animals. There were 31 unique OR clusters in *Ailuropoda melanoleuca*, and 22,107 unique OR gene clusters in *Tremarctos ornatus* and *Ursus* (Figure 5). The specificity of OR gene subfamilies in each species showed that their OR gene families significantly increase the diversity of gene sequences during the evolution process.

### 3.3. Patterns of Conserved Motifs for OR Genes

**Figure 6:**
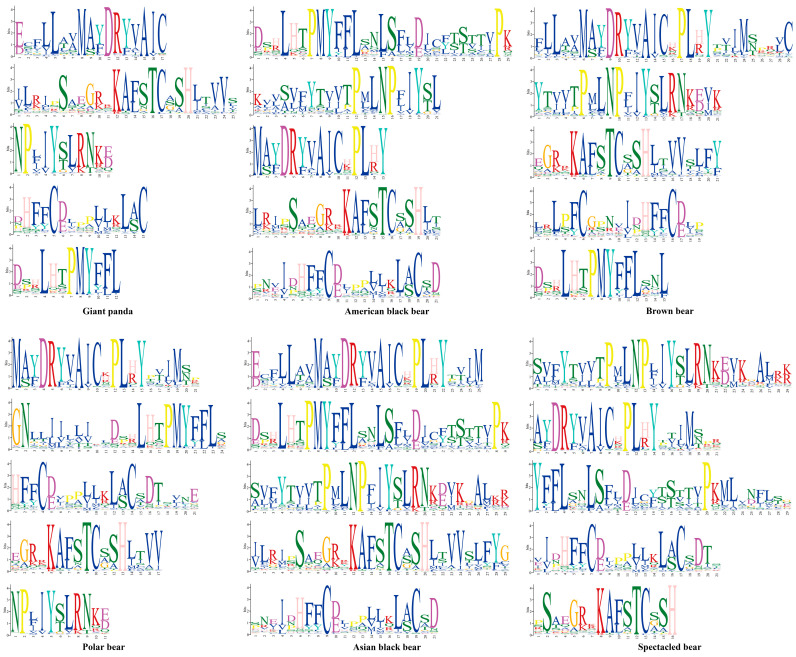
The five most conserved motifs of functional OR genes in six animals. The high degree of amino acid coding represents the degree of conservatism.

Several major features of OR genes include the absence of introns in the CDS region, protein sequence conservation and seven conserved transmembrane domains [4]. In order to test the conservation of OR gene protein sequences, the five most conserved motifs of these six species (giant panda, spectacled bear, American black bear, brown bear, polar bear, and Asian black bear) were identified through the MEME program (Figure 6). Despite the differences of environments in which the six species lived, the composition and location of OR genes were still highly conserved, which was represented by the height of the amino acid code. A conserved N-linked glycosylation site was found in most OR functional genes. The presence of conserved motifs and N-linked glycosylation sites both indicated that OR genes had similar functions at the protein level. Mammalian OR genes usually exhibit single-exon characteristics because there are no introns in the coding region. However, there are untranslated exons upstream of the coding region. Therefore, no matter how many transcripts are spliced during expression, the same protein is obtained after translation [30].

### 3.4. Potential Odorant Specificity of OR Subfamilies

By comparing the protein sequences of functional OR genes with those of human OR genes that previously described odor specificity, we studied the potential target specificity of OR gene subfamilies in odor perception [31]. A total of 220 functional OR genes (giant panda: 51 functional OR genes, spectacled bear: 29 functional OR genes, American black bear: 88 functional OR genes, brown bear: 47 functional OR genes, polar bear: 70 functional OR genes, and Asian black bear: 74 functional OR genes) were matched to known specific human OR genes with at least 60% amino acid sequence identity, indicating that these OR genes may have similar olfactory specificity (Table 3). The sequences similar to OR2B11, OR10G3, OR11H6 and OR11H7P were not found in the giant panda, but were present in five other species. OR2B11 is known to be related to the recognition of coumarin [32]. There is a study showing that coumarin was found in some fruits, such as bilberry and cloudberry [33]. Moreover, OR10G3 is considered to be related to the recognition of vanillin. Vanillin is mainly found in vanilla. It has also been shown to be present in some fruits [34,35]. In addition, OR11H6 and OR11H7P can recognize isovaleric acid [36]. Moreover, isovaleric acid was found in apples [37]. As an obligate bamboo feeder, the giant pandas rarely eat fruits in the wild, which may explain the lack of the genes similar to OR2B11, OR10G3, OR11H6 and OR11H7P in the giant panda.

## 4. Discussion

Here, the giant panda genome we used was 2.29 Gb, which was an increase of 0.04 Gb compared to the previous version of the genome, which filled in about 80% of the gap. We believe that we have identified the OR genes of the giant panda more comprehensively. Of course, due to the gap of 0.01 Gb in the existing sequence, some OR genes may not have been identified. Higher quality of genomes could improve the accuracy of OR gene identification. Thus, the results of the OR gene detection in our study need further verification based on better quality genomes. The OR gene family is the largest multigene family in vertebrates, and they are usually distributed in clusters on the chromosomes. This is the first time that the OR gene of the giant panda has been mapped to the chromosomes. Obtaining basic information about the OR gene family of the giant panda will help to study the olfactory system of this endangered species further.

### 4.1. Olfaction and Transformation of Feeding Habits

About 99% of the food of giant pandas is bamboo, and they eat more than 50 kinds of bamboo. Many previous studies have also been conducted on the change of its eating habits. Genes encoding digestive enzymes, protease, amylase, lipase, cellulase, lactase, invertase and maltase were found in the giant panda genome [38], indicating that the giant panda may have all components required for the digestive system of meat, but no homologues of the cellulase gene have been found. The giant panda’s bamboo eating habits may be more dependent on its gut microbiota rather than its own genome composition [38]. At the same time, the *T1R1* gene of the giant panda has become a pseudogene, which may be the reason why the giant panda is not sensitive to the umami taste of meat and amino acids. Genomes of the giant panda, human, polar bear, ferret, dog, cat, tiger and mouse were compared and analyzed, which revealed that a single substitution from C to T in the 16th exon was found in the giant panda’s *DUOX2* (dioxidase 2) gene, resulting in a premature stop codon (TGA) [39]. It is homologous to human *DUOX2* gene, which is responsible for catalyzing the conversion of water into hydrogen peroxide and encodes a transmembrane protein that is used in the final step of T4 and T3 synthesis. This mutation was also observed in the transcriptome data, indicating that the *DUOX2* transcript would not be translated into a complete protein. In humans and mice, *DUOX2* loss-of-function mutations can lead to hypothyroidism. It may be one of the reasons for the low energy metabolism of the giant panda [39]. Genomes of the red panda (*Aliurus fulgens*) and the giant panda, which all feed on bamboo and have pseudothumbs adapted to eat bamboo, were compared and analyzed [40]. At the genome level, the umami taste receptor gene *TAS1R1* of these two species was pseudogene, which may make them insensitive to meat. At the same time, the limb development genes *DYNC2H1* and *PCNT* have undergone adaptive convergence and may be important candidate genes for the development of pseudothumbs [40].

Olfaction is critical to vertebrates in many aspects, such as food localizing, social communication and mating behavior [41]. Indeed, the olfactory system is essential for the giant panda to choose food and reproduce. Giant pandas mainly rely on their olfaction to determine the nature of food when eating, especially at night, because they are more active at night than during the day. Vision cannot be fully developed at night, and a keen olfaction plays a decisive role in the search and selection of food. Its well-developed olfactory function and poor vision may be related to its limited vision and specialized olfactory sense when it has lived in the dark and dense forest for a long time. The giant panda’s odor-binding protein (OBP) library was identified, and the protein expression was mapped in nasal mucus and saliva using proteomics technology [18]. By comparing the captured data with the structure of bamboo volatiles and the structure of typical mammalian pheromones, they proposed the hypothesis of the chemical pheromone that may be most relevant to the giant panda. Here, we first characterized the OR gene pool in the giant panda based on the complete genome at the chromosome level and compared it with other species. The results showed that the percentage of OR pseudogenes of the giant panda were higher than other five species of the same family Ursidae. The pseudogenes may be related to the identification of meat and fruit, which leads to the changes in eating habits in the giant panda. Clustering analysis showed that the giant panda has 8 unique OR gene families and thirty-one unique OR gene subfamilies among the six Ursidae species, indicating that it may have some special olfactory abilities. Although the giant panda belongs to the Carnivora order, 99% of their food is bamboo. The conversion of their diet may be related to the giant panda’s dependence on smell when foraging, and whether this is related to its unique OR genes needs further verification.

### 4.2. Pseudogenization of OR Genes

During the evolution of OR genes, most OR genes of the same evolutionary branch are located in the same genome cluster. This phenomenon indicates that gene tandem replication is an important molecular mechanism in the evolution of OR genes through gene duplication, gene conversion and other genomic event realization [10]. The number of functional genes reveals to a certain extent the selection pressure that OR genes can withstand in the evolutionary process. If the OR gene becomes more and more important in the evolutionary process, the greater the selection pressure it will bear, leading to the rapid increase in the number of OR genes, and the evolutionary phenomenon of “birth” appears. On the contrary, the evolutionary phenomenon of “destroy” appears in the gene, and the functional gene evolves into a pseudogene [42]. Severe pseudogenization is a distinctive feature of the OR gene family. OR pseudogenes in different mammals are non-functional residues formed during the evolution of gene families. Compared with functional genes, pseudogenes have different degrees of insertion, deletion, etc. Although the gene sequence is very similar to the coding gene, the transcription function is lost. A variety of factors may lead to the pseudogenization of OR genes: frameshift mutations, nonsense mutations, etc., destroy the original coding region; deletion of some conserved sites leads to inactivation of protein function; single nucleotide polymorphisms may change an amino acid at a key position causing the loss of OR’s ability to bind to specific odor molecules; the destruction of the promoter leads to gene inactivation [43].

Pseudogene ratios are different in the OR genes of different species. The main reasons for this phenomenon are as follows. Firstly, the ratio of pseudogenes in OR is affected by the age and disease of the species: the younger and the healthier the species is, the lower the ratio of OR pseudogenes is. Secondly, there is a sensory compensation mechanism between olfactory tissues and other sensory organs. For example, the vision evolution first mechanism will lead to relaxation of OR gene evolution selection pressure [44]. In some primates, the number of OR functional genes is much smaller than that of other mammals, which is consistent with the vision evolution first mechanism of primates. Thirdly, the ratio of OR pseudogenes is related to the particular lifestyle of the species. The platypus, as an amphibian, finds food through electrical signals and tactile perception in the process of foraging. Therefore, its Class-II OR genes have been pseudogenized [45]. The pseudogenes of the giant panda account for about 21.44%, and the proportion of pseudogenes of the other five species is between 11.99% and 18.04%. Therefore, the percentage of pseudogenes on the giant panda was the highest, which may be related to the bamboo-eating specificity. The OR genes of different species have different degrees of pseudogenization in evolution.

### 4.3. Olfactory and OR Functional Genes

The olfactory sensitivity of mammals is positively correlated with the number of OR functional genes [46]. For example, the number of OR functional genes in dogs is twice that of human [45]. The regression of olfactory function caused by the loss of gene function is reflected in different species. For example, human OR pseudogenes account for about 52% of the total number of OR genes, while rat OR pseudogenes account for only about 28% [6,47]. This high pseudogenization is also consistent with the fact that humans’ reliance on smell is far lower than that of non-primates. Mice have 1500 OR genes, and humans have nearly 1000 OR genes. Moreover, the number of OR functional genes in the mouse is about 3 times that of humans [48]. Therefore, in the genome of a species, the number of OR functional genes reflects the sensitivity of the species’ olfaction to a certain extent. The Asian black bear had 792 OR genes and 608 functional OR genes, which was the highest among the six species, indicating that the Asian black bear’s olfaction may be more developed. The Asian black bear has a complex diet, so its survival depends on a keen sense of smell. The diet habit of the giant panda is specific. About 99% of the food of giant pandas is bamboo, which could explain why the percentage of OR functional gene was only 63.85% to some extent. The specificity of food may have contributed to the deterioration of giant pandas’ olfaction.

## 5. Conclusions

We used the chromosome-level genome of the giant panda to identify OR genes and distinguish functional genes, partial genes and pseudogenes. OR genes were compared among different species from the family Ursidae to understand the characteristics of the giant panda OR gene family. The potential odor specificity of some giant panda OR genes was identified by the similarity with human protein sequences. We analyzed the classification and conservative motifs of giant panda OR genes. Comparison of OR genes between species showed that the number and percentage of pseudogenes in the giant panda were both highest, suggesting that the olfaction of the giant panda may be more undeveloped. It will lay a solid foundation for us to further study the relationship between giant panda OR genes and food selection, reproduction and other behaviors.

## Figures and Tables

**Table 1:** Summary of the olfactory receptor (OR) genes in six Ursidae species.

Species	Functional OR Genes		Partial OR Genes		OR Pseudogenes		Total Number of OR Genes	No. of Subfamilies
	Number	Percentage	Number	Percentage	Number	Percentage		
Giant panda (*Ailuropoda melanoleuca*)	408	63.85%	94	14.71%	137	21.44%	639	248
Spectacled bear (*Tremarctos ornatus*)	269	60.04%	106	23.66%	73	16.29%	448	205
American black bear (*Ursus americanus*)	497	80.55%	17	2.76%	103	16.69%	617	249
Brown bear (*Ursus arctos*)	423	72.68%	70	12.03%	89	15.29%	582	241
Polar bear (*Ursus maritimus*)	394	75.62%	33	6.33%	94	18.04%	521	252
Asian black bear (*Ursus thibetanus*)	608	76.77%	89	11.24%	95	11.99%	792	271

**Table 2:** Composition of OR genes for each chromosome of the giant panda.

Location	Chromosome Size (Mb)	No. of Functional OR Genes	No. of Partial OR Genes	No. of OR Pseudogenes	Total Number of OR Genes	No. of OR Subfamilies
Chr1	212.77	12	2	2	16	10
Chr2	199.81	6	0	3	9	5
Chr3	147.63	9	0	4	13	9
Chr4	147.79	15	0	3	18	14
Chr5	130.99	6	1	3	10	6
Chr6	131.59	4	0	0	4	4
Chr7	141.53	13	0	2	15	11
Chr8	129.35	36	1	7	44	36
Chr9	103.69	0	0	0	0	0
Chr10	110.58	4	0	0	4	3
Chr11	110.51	0	0	0	0	0
Chr12	81.78	4	0	0	4	2
Chr13	92.46	6	0	0	6	4
Chr14	106.65	0	1	0	1	0
Chr15	91.61	4	0	1	5	4
Chr16	91.34	30	1	7	38	25
Chr17	42.25	6	0	2	8	6
Chr18	38.12	3	0	1	4	3
Chr19	35.68	0	0	0	0	0
Chr20	30.94	5	0	5	10	5
ChrX	112.85	7	1	5	13	7

**Table 3:** Potential associations between Ursidae OR gene clusters and odorant recognition.

Human ORs	Accession Number	No. of Functional OR Genes						Recognized Odorant(s)
		AIL	AME	ARC	MAR	THI	TRE	
OR1A1	Q9P1Q5	1	1	0	0	1	1	(S)-(−)-citronellol, (S)-(−)-citronellal, (+)-carvone [19]
OR1A2	Q9Y585	0	1	0	1	0	0	Same as OR1A1 except (S)-(−)-citronellol
OR1D2	P34982	2	1	1	1	2	0	Bourgeonal
OR1E3	Q8WZA6	1	1	1	1	0	2	Acetophenone
OR1G1	P47890	1	1	0	0	1	1	Nonanal, 1-nonanol, 2-ethyl-1-hexanol, γ-decalactone, Ethyl isobutyrate, Isoamyl acetate
OR2A25	A4D2G3	1	0	0	0	4	0	Geranyl acetate
OR2AG1	Q9H205	0	1	1	1	0	0	Amylbutyrate
OR2B11	Q5JQS5	0	2	2	2	4	2	Coumarin
OR2C1	O95371	2	0	2	2	2	3	Nonanethiol, Octanethiol
OR2J2	O76002	0	1	0	1	0	0	1-heptanol, 1-octanol, 1-nonanol, 1-decanol, Coumarin, Ethyl vanilin, cis-3-hexen-1-ol
OR2J3	O76001	0	1	0	1	0	0	cis-3-hexen-1-ol, Geranyl acetate, Cinnamaldehyde
OR2M7	Q8NG81	2	5	1	3	2	1	Geraniol (−)-β-citronellol
OR2W1	Q9Y3N9	1	0	0	3	1	1	allyl phenyl acetate, cis-3-hexen-1-ol, Citral and citronellal [19]
OR3A1	P47881	0	11	0	1	0	0	Helional, Lilial
OR4D6	Q8NGJ1	1	1	0	0	1	0	β-ionone
OR4D9	Q8NGE8	1	0	3	2	0	0	β-ionone
OR4Q3	Q8NH05	0	2	0	2	0	0	Eugenol
OR5A1	Q8NGJ0	0	0	0	1	1	0	β-ionone
OR5A2	Q8NGI9	0	0	0	1	1	0	β-ionone
OR5AN1	Q8NGI8	5	1	2	0	1	0	Muscone
OR5D18	Q8NGL1	1	0	1	0	1	0	Eugenol, isoeugenol
OR5K1	Q8NHB7	4	8	5	8	2	2	Eugenol methyl ether
OR5P3	Q8WZ94	1	1	1	1	1	1	1-hexanol, 1-heptanol, (−)-carvone, (+)-carvone, Acetophenone, Coumarin, 1-octanol and celery ketone
OR6P1	Q8NGX9	1	1	1	1	1	1	Anisaldehyde
OR7C1	O76099	3	9	5	5	19	1	Androstadienone
OR7D4	Q8NG98	1	1	0	1	1	1	Androsterone, Androstadienone
OR8B3	Q8NGG8	1	0	1	1	2	0	(+)-carvone
OR8D1	Q8WZ84	1	2	3	1	3	0	Caramel furanone
OR8K3	Q8NH51	1	0	1	0	0	1	(+)-menthol
OR10A6	Q8NH74	1	2	3	1	3	0	3-phenyl propyl propionate
OR10G3	Q8NGC4	0	4	2	2	2	1	Ethyl vanillin, Vanillin
OR10G4	Q8NGN3	1	1	1	0	0	0	Guaiacol, Vanillin
OR10G7	Q8NGN6	1	1	1	0	0	0	Eugenol
OR10G9	Q8NGN4	1	1	1	0	0	0	Ethyl vanillin
OR10J5	Q8NHC4	1	2	2	2	2	2	Lyral
OR11A1	Q9GZK7	0	0	0	0	1	1	2-ethyl fenchol
OR11H4	Q8NGC9	5	7	3	5	4	2	Isovaleric acid
OR11H6	Q8NGC7	0	5	1	5	2	2	Isovaleric acid
OR11H7P	Q8NGC8	0	5	1	5	2	2	Isovaleric acid
OR51E1	Q8TCB6	0	0	0	1	0	0	Nonanoic acid, Butyl butyryllactate, Butyric acid, Isovaleric acid, Propionic acid
OR51E2	Q9H255	0	0	0	1	0	0	Propionic acid
OR51L1	Q8NGJ5	4	1	1	0	0	1	Hexanoic acid, Allyl phenyl acetate
OR52D1	Q9H346	2	1	0	1	4	0	Ethyl heptanoate, Methyl octanoate, 1-nonanol, 2-nonanol, 3-nonanone, 3-octanone
OR56A1	Q8NGH5	1	2	0	2	1	0	Undecanal
OR56A4	Q8NGH8	1	2	0	2	1	0	Decyl adehyde, Undecanal
OR56A5	P0C7T3	1	2	0	2	1	0	Undecanal

AIL: giant panda; AME: American black bear; ARC: brown bear; MAR: polar bear; THI: Asian black bear; TRE: spectacled bear.

## Data Availability

All genome data were downloaded from NCBI (https://www.ncbi.nlm.nih.gov/, accessed on 1 January 2022) (giant panda: GCF_002007445.1, spectacled bear: GCA_018398825.1, American black bear: GCF_020975775.1, brown bear: GCF_003584765.2, polar bear: GCF_000687225.1, and Asian black bear: GCA_009660055.1).
